# Effects of Acute Photobiomodulation on Heart Rate Variability in Physically Active Individuals: A Randomized and Controlled Clinical Trial

**DOI:** 10.1002/jbio.70242

**Published:** 2026-03-11

**Authors:** Reobbe Aguiar Pereira, Aparecida Maria Catai, Juliana Cristina Milan‐Mattos, Adriana Keila Dias, Nivaldo Antonio Parizotto

**Affiliations:** ^1^ Post‐Graduation Program in Biomedical Engineering University Brazil São Paulo São Paulo Brazil; ^2^ Physical Therapy Department Federal University of São Carlos São Carlos São Paulo Brazil

**Keywords:** cardiac autonomic modulation, heart rate variability, photobiomodulation, strength training, vagal neuromodulation

## Abstract

Photobiomodulation (PBM) therapy is widely investigated for tissue recovery, performance enhancement, and post‐exercise optimization. This study assessed whether PBM applied to the vagus nerve in the infra‐auricular region, combined with resistance exercise, modulates cardiac autonomic function in healthy, physically active adults. In this acute randomized controlled trial, 34 volunteers were enrolled: 17 underwent PBM or sham PBM in a crossover design, while a control group (*n* = 17) received PBM without exercise. PBM was delivered with a total energy dose of 12 J. Heart rate variability (HRV) was analyzed in time, frequency, and non‐linear domains. Results showed a slight reduction (*p* = 0.011) in approximate entropy (ApEn) in the PBM group compared with controls, but no significant differences were observed in other HRV indices. These findings suggest that PBM applied to the vagus nerve induces minimal acute modulation of autonomic complexity. Further studies with different dosimetries and exercise protocols are warranted.

## Graphical abstract

This article is the first to approach the use of light acting on the vagus nerve in individuals undergoing physical exercise. Although we only obtained one isolated parameter as a difference in HRV, it is a first indication that we may be able to intervene in this vagal control process over the heart with PBM. Future perspectives are shown for new clinical trials with different doses and wavelengths to analyze the possibility of more robust effects.

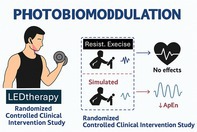

## 1 Introduction

Photobiomodulation (PBM), also known as low‐level laser therapy, has been the subject of increasing interest in the scientific community due to its potential modulatory effects on the autonomic nervous system (ANS) [1]. In particular, the application of PBM in the inferior ganglion of the vagus nerve emerges as a promising strategy to influence heart rate variability (HRV) based on another kind of energy application like electrical stimulation [2].

HRV is considered an interesting tool to assess cardiac autonomic modulation. Thus, HRV can be described by the oscillations of the intervals between consecutive heartbeats (R‐R intervals) [3], which also establish anatomical relationships and establish influences of the ANS on the sinus node. It is worth noting that HRV is a non‐invasive measurement, widely used to identify phenomena related to the ANS in healthy individuals [4, 5], as well as athletes and those with diseases [6]. Individuals who undergo acutely strength exercises often present changes in HRV, characterized by a reduction in parasympathetic influence and an increase in sympathetic activity [7].

Cardiac autonomic modulation by electrophysical agents has been studied, highlighting several therapeutic approaches. Electrical stimulation of the vagus nerve (VNS) is a technique that has shown promising results in the treatment of cardiac arrhythmias and heart failure. Studies indicate that VNS can modulate autonomic activity, promote antiarrhythmic effects and improve cardiac function [8] also showed potential as an additional treatment for patients with Covid‐19 [2, 9]. Other therapeutic modalities, such as tragus stimulation, renal denervation (RDN), baroreceptor stimulation (BAT) and cardiac sympathetic denervation (CSD), have also been explored for their potential to modulate autonomic activity and treat arrhythmias [8].

PBM is a technique that uses light (LED/LASER) at specific wavelengths (red and near infrared) to induce photobiological effects in tissues. Studies suggest that PBM can influence autonomic modulation, including cardiovascular activity [10]. There are already devices developed to provide non‐invasive stimulation of the vagus nerve through PBM, aiming to improve brain‐body connectivity and regulate autonomic functions [11]. In addition, they explored the use of PBM to stimulate specific acupuncture points, such as *Neiguan* (PC6) and *Shenmen* (HT7), with the aim of modulating the ANS, affecting HRV and sympathetic and parasympathetic activity [12]. Although initial results are promising, more research is needed to fully understand the mechanisms and establish effective clinical protocols for the application of PBM in cardiac autonomic modulation. The specific application of PBM in the inferior ganglion of the vagus nerve in individuals who perform strength exercises is a developing field, as the existing literature suggests that interventions aimed at autonomic modulation may have beneficial effects on HRV and, consequently, on cardiovascular health [10, 11]. Therefore, there is no evidence to date on the effect of PBM applied to the vagus nerve in combination with resistance exercise on HRV.

The aim of the study was to evaluate the influence of the acute application of PBM therapy in the auricular emergence of the vagus nerve, in association with a physical exercise protocol, on cardiac autonomic modulation in apparently healthy physically active individuals of both sexes.

## 2 Materials and Methods

### 2.1 Study Design

A randomized, crossover, controlled, simple blind, acute intervention clinical trial.

### 2.2 Ethical Aspects

The research was approved by the Human Research Ethics Committee (CEP) of Universidade Brasil, São Paulo, Brazil (#5.059.309) and registered in the Brazilian Clinical Trials Registry (REBEC) **RBR‐5tkh5hv** Effects of Photobiomodulation on HRV responses in bodybuilders.

### 2.3 Sample

The sample consisted of individuals aged between 18 and 50 years, of both sexes, who practiced bodybuilding for at least 3 months and who were registered in the e‐SUS system at the Academia da Saúde—Almir Coelho Filho, in the city of Guaraí, in the state of Tocantins, Brazil.

Individuals with neurological and muscular diseases; multiple system atrophy; pain or limitation in movement of the upper or lower limbs and who have suffered some type of serious injury in the last 6 months; chronic systemic diseases, for example, diabetes mellitus, systemic hypertension, cardiovascular, renal, cerebral diseases, neoplasms or HIV‐AIDS, and cognitive inability to understand or communicate significantly were excluded of the study used as exclusion criteria.

The sample size calculation was performed based on the sample size determination using the G‐Power program version 3.1.9.4 (https://g‐power.apponic.com), adopting a standard significance level of 5% (*p* < 0.05), with power 80% (0.80), obtaining the number of 17 volunteers per group, used HRV as the dependent variable with a repeated measures ANOVA test based on a previous study by the group [13].

This is a crossover study, and the randomization process was performed using sealed envelopes with an order generated on the computer using Microsoft Excel, following the CONSORT 2010 requirement. For this reason, the group was exposed to two types of treatment (active and placebo). We used 1 week of washout period to avoid possible residual effects. In addition, there was a control group (CL) that served to observe the effects of PBM on the untrained individual, to evaluate the effects outside the exercise environment. Blinding was performed with protective glasses with which the individuals did not know whether they were currently in the active or placebo group (using the equipment turned off).

The participants performed the experimental protocol of PBM therapy using red LED (660 nm), before practicing the weight training exercise program, in two sessions for 1 week (PBM). After the end of this protocol, a 15‐day washout period was expected, and the protocol was restarted. At this stage, the participants received placebo PBM therapy (PBMP), in which case the equipment was turned off so that it would not provide the electromagnetic wave, after practicing the exercise program, in two sessions for 1 week, on the same days as the training. The individuals did not know which moment (active or placebo) they were in because they were blinded by protective glasses that completely blocked their vision.

LED Control Group: (CL, *n* = 17) the individuals in this group were not subjected to the exercise program, but only to the red LED effect, with the same parameters. Being verified at rest with the same time (in minutes) as the group that performed the exercises.

### 2.4 Experimental Protocol

The participants were all evaluated at the same time of day, considering any circadian influences on their biological cycles. Before the start of the protocol, participants were instructed about the procedures and activities in which they would participate and were advised not to perform strenuous exercises and not to consume alcoholic beverages and stimulants (tea, coffee, soda, chocolate, and energy drinks) the day before and on the day of the tests [14].

On the day of the experiment, the heart rate (HR) monitor (Polar H10) was attached to the participant's chest. This equipment detects the R wave of the electrocardiogram and calculates the distance between consecutive R waves. The values of the R‐R intervals were stored and later transferred to the computer for later analysis of HRV.

**FIGURE 1:**
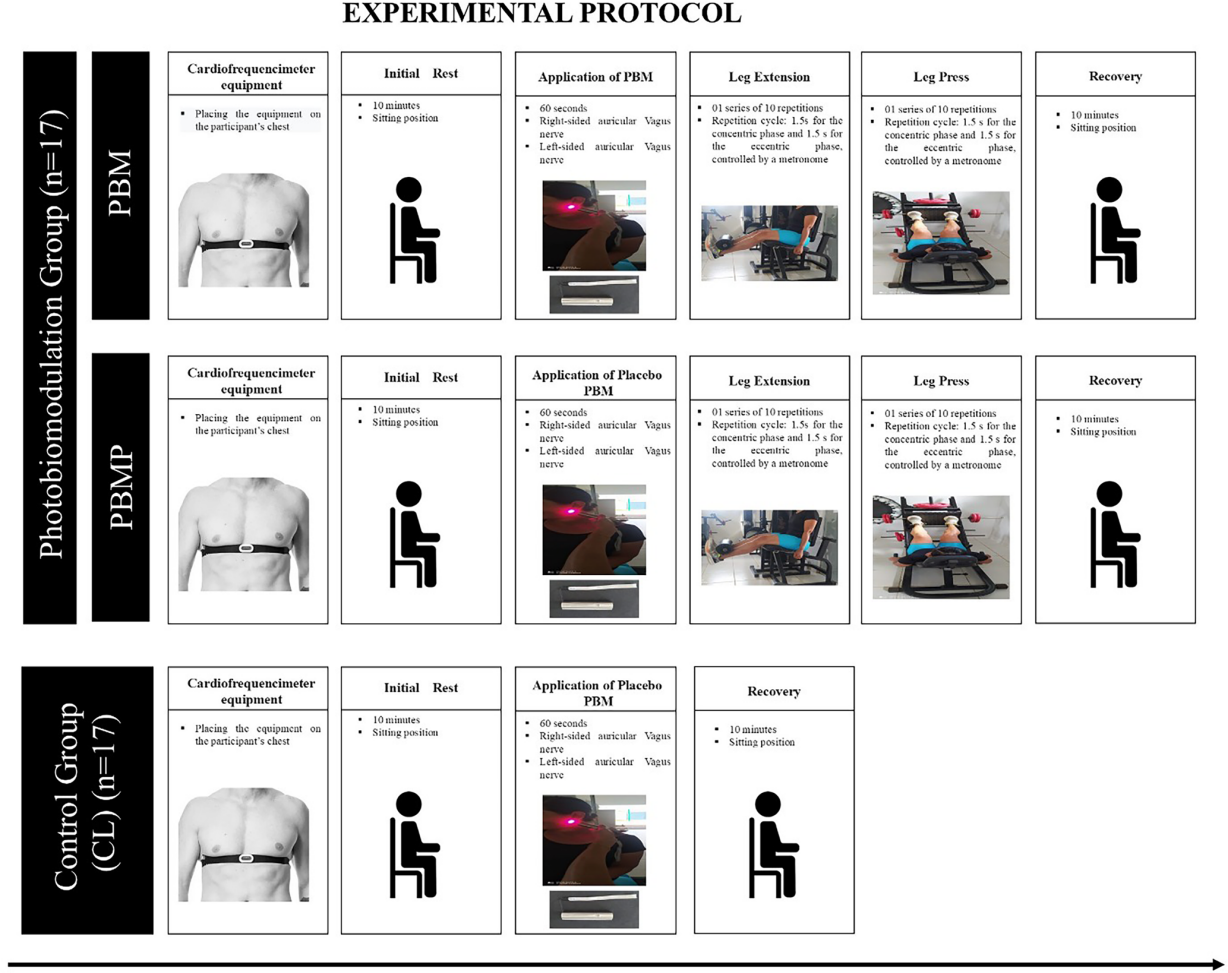
Details of the experimental protocol containing the placement of the heart rate monitor, the initial rest steps, application of PBM, the exercises performed and the recovery period.

The experimental protocol began with a period of 10 min of sitting rest (Initial Rest), then PBM was applied to the vagus nerve, which emerges just below the auricle, anterior to the carotid artery, for 60 s on each side. Subsequently, the individuals underwent weight training on the leg extension chair and leg press, with 01 series of 10 repetitions, with each repetition cycle lasting 3 s, with 1.5 s for the concentric phase and 1.5 s for the eccentric phase, controlled by a metronome: with a knee movement angle between 90 degrees and 180 degrees. To finish, the participants were instructed to sit down and remain at rest for another 10 min (Recovery) (Figure 1).

During the resting protocol (Initial Rest and Recovery), the participants were instructed not to speak unnecessarily and to avoid moving.

The PBM was applied using the Tendlite Medical Device (San Diego, CA‐USA) using LED with 215 mW of power output and spot area of 2.54 cm^2^, wavelength in the red spectral range (660 nm) and using a dose of 5.07 J/cm^2^, for 60 s, in the infra‐auricular vagus nerve emerging region (Table 1). Considering the two points of vagus nerve emergency application, it was delivered 25.8 J of total energy per session (12.9 J per side). Prior to the start of the experimental collections, a pilot analysis was performed to check the safety and applicability, but to date, no deleterious or side effects under the action of PBM have been observed in our analysis and in the literature.

**TABLE 1:** Parameters used in the protocol with LED‐PBM.

Parameters	Values
Power (mW)	215 mW
Wavelength (nm)	660 nm
Local and frequency of applications	Vagus nerve infra‐auricular bilateral—2 sessions per week
Spot area (cm^2^)	2.54 cm^2^
Energy Density (J/cm^2^)	5.07 J/cm^2^
Time (s)	60 s
Total energy per session (J)	25.8 J

Abbreviations: cm^2^, square centimeter; J/cm^2^, Joule per square centimeter; J, Joule; mW, miliWatt; nm, nanometer; s, seconds.

### 2.5 Data Analysis

For the HRV analysis, the Kubios HRV Standard 3.5.0 Software [33, 34] was used. All files were carefully checked for possible artifacts. For this, a filter available in the software was used for correction, following the criteria for signal cleaning previously described in the literature [3, 14].

Five‐minute stable sections were chosen in the initial rest and recovery positions, where linear and non‐linear analyses were evaluated.

The linear indices were evaluated in the time domain, namely: Mean; standard deviation; minimum and maximum HR value; mean of the R‐R intervals; standard deviation of the mean of the iRR (SDNN) and the square root of the mean differences between successive iRR (RMSSD), an index related to parasympathetic modulation [14].

And in the frequency domain, namely: spectral component in low (0.04–0.15 Hz) (LF, ms2) and high frequency (HF, ms2), in absolute units, which represent mixed and parasympathetic modulation, respectively. Spectral component at low (0.04–0.15 Hz) (LFun) and high frequency (HFun) in normalized units, representing sympathetic and parasympathetic modulation, respectively. Relationship between the powers of the LF and HF bands in absolute units (LF/HF), representing the sympathovagal balance [15, 16].

To complement the linear analysis, the following nonlinear indices were analyzed. Poincaré plot: Standard deviation perpendicular to the identity line (SD1), representing parasympathetic modulation; Standard deviation along the global variability line (SD2), representing mixed modulation; and the relationship between the SD1 and SD2 indices (SD2/SD1) [17]. Approximate (ApEn) and sample (SampEn) entropy: Both quantify the regularity/complexity of the fluctuations in the iRR series; the higher the value, the greater the regularity/complexity of the series [18]. Detrended Fluctuation Analysis: short‐term fluctuations (Alpha 1) and long‐term fluctuations (Alpha 2) [19].

The linear indices were evaluated in the time domain (Mean; standard deviation; minimum and maximum HR value; mean of the R‐R intervals; standard deviation of the mean of the iRR (SDNN) and the square root of the mean differences between successive iRR (RMSSD)), frequency domain (spectral component in low (0.04–0.15 Hz) (LF, ms2) and high frequency (HF, ms2), in absolute units, spectral component at low (0.04–0.15 Hz) (LFun) and high frequency (HFun) in normalized units and relationship between the powers of the LF and HF bands in absolute units (LF/HF)). Nonlinear indices were analyzed (Poincaré plot: Standard deviation perpendicular to the identity line (SD1); standard deviation along the global variability line (SD2), representing mixed modulation; and relationship between the SD1 and SD2 indices (SD2/SD1); Approximate (ApEn) and sample (SampEn) entropy; Detrended Fluctuation Analysis: short‐term fluctuations (Alpha 1) and long‐term fluctuations (Alpha 2)).

### 2.6 Statistical Analysis

The statistical analysis was performed using the statistical program (SigmaPlot 11.0, Systat, Chicago, IL, USA). With a significance level of *p* < 0.05. The Shapiro–Wilk test was used to test the normality of the data. To compare age and anthropometric data, the Student's *t*‐test or Mann Whitney test was performed, depending on the normality of the data. To compare the deltas (Δ recovery—initial rest) between the three studied groups, the One‐Way Analysis of Variance or Kruskal‐Wallis One Way Analysis of Variance on Ranks (Tukey's post hoc) test was used, depending on the normality of the data. The Pearson (normal distribution) or Spearman (non‐normal distribution) correlations between ApEn and sympathetic and parasympathetic indexes were done in the studied groups.

## 3 Results

A total of 34 participants were included, 17 in the PBM/PBMP and 17 in the CL groups were evaluated. Table 2 shows the age, sex, and anthropometric data of the study participants. It is important to remember that the experimental and placebo groups are the same group of participants, as it is a randomized, cross‐over study; after a washout period, they were reused for new analysis.

**TABLE 2:** Age, sex, and anthropometric data of study participants.

	CL (*n* = 17)	PBM/PBMP (*n* = 17)
Age (years)	32 ± 11	37 ± 09
Sex	12F/5H	12F/5H
Anthropometric data		
Height (m)	**1.69 ± 0.08**	**1.63 ± 0.11** ^**a**^
Body Mass (Kg)	70.31 ± 10.44	64.68 ± 10.44
BMI (Kg/m^2^)	24.65 ± 2.50	24.37 ± 2.93

*Note:* Data expressed as mean ± standard deviation.

Abbreviations: BMI, body mass index; CL, Control group; PBM, Photobiomodulation active treatment with exercise group; PBMP, Photobiomodulation placebo treatment with exercise.

^a^ *p* = 0.035.

The groups were similar in terms of age, sex, body mass, and BMI. Height was different between the groups, with the CL showing the highest value.

Table 3 shows the comparison of the delta (recovery—initial rest) of the HRV indices, considering the three groups studied (CL, PBM, PBMP).

**TABLE 3:** Comparison of the delta of HRV indices in the studied groups.

	CL (*n* = 17)	PBM (*n* = 17)	PBMP (*n* = 17)
Linear analysis			
Time domain			
Δ Mean IRR (ms)	‐4.59 ± 36.94	17.00 ± 40.94	8.71 ± 41.42
Δ SDNN (ms)	−3.03 ± 11.39	−0.05 ± 11.42	3.46 ± 16.63
Δ Mean HR	0.05 ± 3.57	−1.32 ± 3.60	−0.45 ± 3.15
Δ Standard Deviation HR	−0.30 ± 1.10	−0.12 ± 0.94	0.15 ± 0.97
Δ Min HR	0.29 ± 4.22	−0.23 ± 3.60	−1.14 ± 3.13
Δ Max HR	1.00 ± 4.01	−0.28 ± 5.36	1.40 ± 4.72
Δ RMSSD	−2.64 ± 8.36	−0.09 ± 11.86	3.97 ± 21.51
Frequency domain			
Δ LF (ms^2^)	−362.57 ± 1233.37	−57.90 ± 694.65	34.14 ± 580.05
Δ HF (ms^2^)	−79.64 ± 328.29	122.54 ± 467.09	444.36 ± 1691.62
Δ LF (nu)	0.41 ± 14.77	1.43 ± 12.61	6.23 ± 11.60
Δ HF (nu)	−0.43 ± 14.76	−1.38 ± 12.53	−6.20 ± 11.59
Δ LF/HF	0.27 ± 3.95	0.40 ± 1.05	0.85 ± 1.03
Nonlinear analysis			
Poincaré plot			
Δ SD1 (ms)	−1.87 ± 5.92	−0.06 ± 8.40	2.81 ± 15.24
Δ SD2 (ms)	−3.73 ± 15.67	−0.31 ± 14.53	3.62 ± 18.45
Δ SD2/SD1	0.02 ± 0.67	0.06 ± 0.35	0.20 ± 0.34
Entropy			
Δ ApEn	**0.04 ± 0.10**	**−0.02 ± 0.05** ^a^	0.02 ± 0.03
Δ SampEn	0.09 ± 0.23	0.00 ± 0.17	0.01 ± 0.10
Detrend fluctuation analysis			
Δ Alpha 1	0.01 ± 0.23	0.03 ± 0.20	0.05 ± 0.19
Δ Alpha 2	0.02 ± 0.08	−0.01 ± 0.16	0.02 ± 0.15

*Note:* Data expressed as mean ± standard deviation.

Abbreviations: Alpha 1, short‐term fluctuations; Alpha 2, long‐term fluctuations; ApEn, approximate entropy; SampEn, sample entropy.

^a^ CL × PBM a = 0.011.

The Δ of the indices of the linear analysis (time and frequency domain) did not show differences between the three groups studied. As for the nonlinear analysis, only the approximate entropy index (ApEn) showed a statistical difference between the CL and the PBM, where the CL showed the highest Δ. This finding allows us to identify that in the CL, where only the PBM was applied, the ApEn showed higher values in the recovery, evidenced by a positive Δ value.

In the PBM, the Δ was negative. Since exercise promotes an increase in sympathetic modulation and, consequently, a reduction in entropy, this may have been the reason why this group showed lower entropy values in the recovery.

Table 4 presents the correlation between ApEn and sympathetic and parasympathetic indexes. In the CL group, no correlations were observed between the ApEn indices and those representing parasympathetic modulation. However, a negative correlation was observed at rest between the ApEn indices and the SDNN index.

**TABLE 4:** Correlations between ApEn and sympathetic and parasympathetic indexes in studied groups (PBM and PBMP).

	ApEn					
	CL		PBM		PBMP	
	Rest	Recovery	Rest	Recovery	Rest	Recovery
SDNN	*R*: −0.50 *p*: 0.004	NS	*R*: −0.505 *p*: 0.038	*R*: −0.596 *p*: 0.012	*R*: −0.687 p: 0.0023	*R*: −0.779 *p* < 0.001
RMSSD	NS	NS	*R*: 0.747 *p*: 0.001	*R*: −0.615 *p*: 0.009	*R*: −0.684 *p*: 0.003	*R*: −0.779 *p* < 0.001
LF (nu)	NS	NS	NS	*R*: 0.690 *p*: 0.002	NS	NS
HF (nu)	NS	NS	NS	*R*: −0.692 *p*: 0.002	NS	NS

Abbreviations: ApEn, Approximate entropy; CL, Control group; HFnu, high frequency in normalized units; LFnu, low frequency in normalized units; *p*, *p* value; PBM, Photobiomodulation active treatment with exercise group; PBMP, Photobiomodulation placebo treatment with exercise group; R: Correlation Coefficient; RMSSD: square root of the mean differences between successive RR‐i; SDNN: standard deviation of the mean of the RR‐i.

For the PBMP group, there was a negative correlation between ApEn and SDNN and between ApEn and RMSSD at rest and during recovery. However, the indices in the frequency domain did not show any correlations.

For the PBM group, there was a negative correlation between ApEn and SDNN at rest and recovery and a positive relationship between ApEn and RMSSD at rest. During recovery, there was a positive correlation with LFnu and a negative correlation with RMSSD and HFnu.

## 4 Discussion

When analyzing the effect of applying PBM therapy to the vagus nerve in the infra‐auricular region in association with a physical exercise protocol, little impact was observed on the cardiac autonomic modulation at rest in bodybuilders. Only one finding was observed in relation to the non‐linear analysis with a statistical difference in the ApEn variable (entropy variation) between the PBM and CL groups. This result can be explained by the effects of performing the exercises and their application in conjunction with PBM (observed in Table 3). This was not observed in individuals who performed PBMP, showing that there was an effect due to the association of PBM with exercises.

Some studies addressed the same topic as the present research, also obtaining similar results. In the study by Milan‐Mattos et al. [10], it was also observed that PBM applied prior to aerobic exercise was not able to modify Baroreflex Sensitivity (BRS) in the time domain; however, the target audience was participants with Diabetes Mellitus.

In this context, for the analysis and understanding of HRV, some indexes obtained through linear methods (such as in the time and frequency domains) are of utmost importance. In addition, there are also non‐linear methods that can be considered. Firstly, the so‐called linear methods can be subdivided into analysis in the time domain, carried out through statistical indexes, and analysis in the frequency domain [3, 7, 14].

Regarding the results, according to Table 3, only the ApEn index presented a positive result, with the CL group having the highest value compared to the active PBM. However, there was no difference with the PBMP group. It is worth noting that the use of LEDs in the red wavelength for phototherapy is important, since this spectral band has good penetration depth and has been shown in several studies to have positive effects on neuromodulation and inflammatory processes, for example [20, 21, 22]. In addition, it is known that LASERs have a monochromatic, coherent, and collimated beam, unlike LEDs, such as the one used in this study, since we must consider the optical properties of biological tissues, which are related to variable rates of absorption, scattering, transmission, and reflection [23]. The study by [24] considered the acute effects of PBM therapy on the local concentration of hemoglobin, assessed by the so‐called near‐infrared spectroscopy (NIRS). The authors analyzed the use of PBM therapy with a light‐emitting diode (850 nm, 50 mW, 2 J), showing an increase in the total concentration of oxymyohemoglobin in the flexor carpi ulnaris muscle of the right or left forearm, selected in random order, which evidenced a balancing effect between the supply and use of oxygen promoted by PBM. Specifically, the authors concluded that the effect of PBM appears to be directly related to the availability of oxygen to the local tissue in an acute (rapid) manner. It is worth noting that PBM therapies can be used to increase oxygen availability, being effective in situations where the target audience has diseases that cause limitations in oxygen supply, such as diabetes, for example. The study by Milan‐Mattos et al. [10] used individuals with Diabetes Mellitus to analyze the effect of PBM performed by LEDs on BRS during and after constant‐load exercises on a cycle ergometer. PBM was applied continuously, using a contact technique, bilaterally to the quadriceps femoris and gastrocnemius muscle groups. Two dosimetry's (150 and 300 J) and placebo were compared, and in this constant‐load exercise protocol, there were no effects in the cardiovascular autonomic control promoted by PBM.

In the study by Flatt et al. [25], which studied HRV together with analyses of neuromuscular recovery and perceptual markers after resistance training with different types of exercises (squat, bench press, and pull‐down), they found variations in the recovery process of these intense exercises, with different recovery times for each of the methods used for evaluation, observed an absence of correlation between them, also showing that HRV was sensitive in this detection. In contrast, in the data from our study, in which moderate exercise was used, these indices did not present changes, with only the index that represents entropy (ApEn) undergoing slight change.

The dosimetry of the LED used may have been another limiting factor, since only one dose was used in this study (6 J on each side, for a total of 12 J), which could be modified in other trials with a larger number of volunteers, and a dose‐response curve could be performed to establish the ideal dosimetric factor for this modulation. Therefore, when the energy applied is insufficient, either due to reduced time or inadequate power density (irradiance), it may result in a lack of effects and therefore not present the expected benefits.

Most elderly people have a higher cardiovascular risk, since changes in autonomic balance can have serious consequences for health [26]. In the present study, the participants are healthy younger individuals who practiced physical activity with regular exercises of moderate nature.

In relation to HR, it is modulated by the sympathetic and parasympathetic systems. Thus, it is understood that HRV is considered a marker of the individual's cardiac autonomic control, being a quantitative index of minimal changes in heartbeats [8, 23]. Thus, while HR refers to the number of times the heart beats per minute, HRV measures the time between each heartbeat. Also known as the R‐R interval, this beat‐to‐beat interval variation is measured in milliseconds and can vary depending on several factors. Furthermore, HRV provides clues about the regulation of the ANS and demonstrates the individual's system's ability to react to stressors, including exercise. In addition, another indicator of the coordination capacity between the sympathetic and parasympathetic nervous systems is also related to this HRV capacity. According to Addleman et al. [27], HRV appears to be a useful metric for assessing the training status, adaptability, and recovery of trained athletes in a variety of sports and physical activities, as well as untrained individuals. Factors that interfere with the strength training process can be tracked by HRV, being a sensitive method for adjusting the exercise intensities imposed on individuals. In this sense, perhaps this was a factor that interfered in the absence of more robust and broader HRV results analyzed in our results. The same rationale can be established for aerobic exercises according to da Silva et al. [28]. HRV was analyzed in elderly individuals, using wearable equipment, to determine the level of physical activity in this population using different observation methods already established in the literature, establishing a correlation between the tests applied in regular assessments of frailty in these individuals, observing the possible correlation between them, having shown itself to be a productive biomarker of these physical functions [29].

Based on the correlation results (Table 4), it was not possible to observe a significant relationship between the ApEn index and the indexes representing parasympathetic modulation in the CL group, except for the SDNN index, which showed a negative correlation at rest.

For the PBMP group, there was a negative correlation between ApEn and SDNN and between ApEn and RMSSD at rest and during recovery. The negative relationship between ApEn and RMSSD is conflicting because the literature indicates that entropy has a positive relationship with parasympathetic indexes; that is, if entropy is high, parasympathetic modulation is also high [30]. The indexes in the frequency domain did not show any relationships.

For the PBM group, there was a negative correlation between ApEn and SDNN at rest and recovery and a positive relationship between ApEn and RMSSD at rest. This finding is consistent with the literature [30, 31]. However, this relationship was not identified in the CL group, which indicates that the finding of the difference in ApEn between the PBM and CL groups may have happened randomly. In recovery, there was a positive correlation with LFnu and a negative correlation with RMSSD and HFnu; these findings do not reflect what is already described in the literature [30, 31].

When analyzing a clinical study like ours, we can consider countless variables, but it is important to focus on certain aspects, such as what we did using an exercise model (in this case, strength), a single PBM dosimetry, and a single HRV approach strategy. When compared with other methods of vagal stimulation, such as in the case of Stavrakis et al. [32], who performed transcutaneous electrical stimulation at an infra‐sensory level, the authors achieved positive and interesting results when applying it in a randomized, sham‐controlled, and double‐blind clinical trial to patients with postural tachycardia syndrome. Therefore, it is necessary to expand the possibilities of future clinical studies to find an adequate PBM dosimetry and maybe other wavelength, as well as the ideal exercise patterns capable of being modulated by the vagus, to obtain a more accurate response.

In summary, we can conclude from the data obtained in this study that PBM with 12 J applied in the vagus nerve in the infra‐auricular region of the active subjects was able to promote changes only in one index of entropy complexity of HRV. However, as this was a pilot trial, it is likely that if we use a dose‐response curve of PBM and eventually other more intense exercise protocols or other strategies, we will have a probability of achieving a modulating effect of PBM in this physiological parameter. Therefore, we suggest further studies with this model of vagal modulation by PBM, which is safe, quite gentle in its application, and has not shown adverse effects.

## Funding

The authors have nothing to report.

## Conflicts of Interest

The authors declare no conflicts of interest.

## Data Availability

The data that support the findings of this study are available on request from the corresponding author. The data are not publicly available due to privacy or ethical restrictions.
